# Calpain 3 is important for muscle regeneration: Evidence from patients with limb girdle muscular dystrophies

**DOI:** 10.1186/1471-2474-13-43

**Published:** 2012-03-23

**Authors:** Simon Hauerslev, Marie-Louise Sveen, Morten Duno, Corrado Angelini, John Vissing, Thomas O Krag

**Affiliations:** 1 Department of Neurology, Neuromuscular Research Unit, The Copenhagen Muscle Research Center, Rigshospitalet, Blegdamsvej 9, Copenhagen, Denmark; 2 Department of Clinical Genetics, University of Copenhagen, Rigshospitalet, Blegdamsvej 9, Copenhagen, Denmark; 3 Department of Neurosciences, University of Padova, Via 8 Febbraio 1848, Padova, Italy; 4 IRCSS S. Camillo, Via Alberoni 7, Venice, Italy

**Keywords:** Limb girdle muscular dystrophy, Calpain 3, Muscle regeneration, INF, Neonatal myosin heavy chain, Vimentin

## Abstract

**Background:**

Limb girdle muscular dystrophy (LGMD) type 2A is caused by mutations in the CAPN3 gene and complete lack of functional calpain 3 leads to the most severe muscle wasting. Calpain 3 is suggested to be involved in maturation of contractile elements after muscle degeneration. The aim of this study was to investigate how mutations in the four functional domains of calpain 3 affect muscle regeneration.

**Methods:**

We studied muscle regeneration in 22 patients with LGMD2A with calpain 3 deficiency, in five patients with LGMD2I, with a secondary reduction in calpain 3, and in five patients with Becker muscular dystrophy (BMD) with normal calpain 3 levels. Regeneration was assessed by using the developmental markers neonatal myosin heavy chain (nMHC), vimentin, MyoD and myogenin and counting internally nucleated fibers.

**Results:**

We found that the recent regeneration as determined by the number of nMHC/vimentin-positive fibers was greatly diminished in severely affected LGMD2A patients compared to similarly affected patients with LGMD2I and BMD. Whorled fibers, a sign of aberrant regeneration, was highly elevated in patients with a complete lack of calpain 3 compared to patients with residual calpain 3. Regeneration is not affected by location of the mutation in the *CAPN3 *gene.

**Conclusions:**

Our findings suggest that calpain 3 is needed for the regenerative process probably during sarcomere remodeling as the complete lack of functional calpain 3 leads to the most severe phenotypes.

## Background

Limb Girdle Muscular Dystrophy type 2A (LGMD2A) is an autosomal recessively inherited disorder characterized by symmetrical progressive proximal muscle weakness and atrophy that usually starts in the pelvic girdle musculature. However, the clinical course is characterized by great variability, ranging from severe forms with onset in the first decade and rapid progression to milder forms with later onset and slower course [1,2]. Serum creatine kinase is usually highly elevated and the muscle morphology shows dystrophic features. The disorder is caused by a loss of functional calpain 3, a skeletal muscle specific isoform of the Ca^2+^-dependent calpain cysteine protease family [3]. More than 460 pathogenic mutations, covering almost the entire length of the calpain 3 gene (*CAPN3*), have been discovered (Leiden Muscular Dystrophy Pages, http://www.dmd.nl). Recent research involving calpain 3 protease-inactive knock-in mice have demonstrated that calpain 3 appears to play a role in the Ca^2+^-efflux from the sarcoplasmatic reticulum in a way that does not involve the protease function of calpain 3, this may explain why dysfunctional calpain 3 leads to muscle weakness [4]. However, the potential substrates of calpain 3 have been a major focus of LGMD2A research in the past few years, as it appears that calpain 3 may be involved in multiple aspects of muscle function and maintenance. Calpain 3 is anchored to the giant structural/scaffold protein titin in a stable and inactive manner, to keep it from degrading itself autolytically [3,5-7]. For that very same reason, the substrates of calpain 3 is believed to be in close proximity, possibly bound to other parts of the sarcomeres. It is thought that calpain 3 is inactive most of the time, only to be activated and redistributed when sarcomeres are exercised beyond a threshold, leading to interaction with a number of proteins e.g. myosin light chain 1, suggesting a role for calpain 3 in sarcomere remodeling [5,8,9]. Another important in vivo calpain 3 substrates that has been described is AHNAK, a very large protein involved in subsarcolemmal cytostructure and part of the dysferlin membrane repair complex, requires calpain 3 for it to be cleaved and the membrane repair to proceed [10]. Hence, it is a key component in the repair of the wear and tear of skeletal muscle tissue. The search for calpain 3 substrates using cleavage site recognition have lead to a number of potential targets, one being Protein Inhibitor of Activated Stats 3 (PIAS3), an ubiquitously expressed E3 SUMO ligase implicated in many signaling pathways by modifying the localization and role in transcriptional regulation of transcription factors [11,12].

A recent study using a protease-inactive calpain 3 knock-in model, demonstrated that calpain 3 lacking protease functionality lead to muscular dystrophy, exacerbated by exercise [5]. This group proposed that the protease activity of calpain 3 is required to protect muscle from degeneration under exercise-induced stress, and that loss of protease activity affected the dynamic distribution of calpain 3 during physical activity and its interaction with MARP2, a stress-response transcriptional regulator protein, in close proximity to calpain 3 on the N2A region of titin. MARP2 is upregulated during exercise in normal muscle, however, the protease-inactivity of calpain 3 resulted in decreased levels of MARP2 and abnormal levels of dysferlin in exercised calpain 3 knock-in mice, leading to the conclusion that the muscle membrane repair mechanism is greatly affected by the loss of calpain 3 protease activity even if the activation of satellite cells is not affected [7]. Several studies have shown that calpain 3 deficiency leads to formation of abnormal sarcomeres, impairment of muscle contractile capacity and loss of the muscle fibers [13,14].

In order to balance the ongoing degeneration, muscle fibers must regenerate, but in patients with LGMD2A it is unknown how calpain 3 deficiency affects the regenerative response. We therefore investigated the level of regeneration in skeletal muscle of 22 patients with genetically confirmed LGMD2A by assessing commonly used markers of muscle regeneration. Internally nucleated fibers (INF) arise from muscle specific stem cells, satellite cells. These cells are activated during muscle degeneration and constitute the majority of regenerative response to muscle wasting. Activation of satellite cells ultimately leads to migration of nuclei to the damaged area of the muscle fiber. This process is evident in the regenerative phase after rhabdomyolysis [15]. For more detailed analysis of muscle regeneration immunohistochemical staining for the developmental myogenic markers, neonatal myosin heavy chain (nMHC), vimentin, MyoD, and myogenin are employed as used in a recent study [16]. MyoD and myogenin is also used as a diagnostic marker for rhadomyosarcomas [17]. These markers are known to be upregulated during myogenesis and muscle fiber regeneration for a short period of time ranging from days to 1-3 weeks [18-22]. Furthermore, we wanted to determine if apoptosis is present in muscle from patients with LGMD2A, as has been suggested previously [14]. The morphology and level of regeneration in patients with two null alleles in CAPN3 were compared with those in patients with limb girdle muscular dystrophy type 2I (LGMD2I) and patients with Becker muscular dystrophy (BMD), who clinically and morphologically resemble patients with LGMD2A [23].

## Methods

### Patients

Twenty-two patients with genetically verified LGMD2A (age 37 ± 14 years) participated in this study described in (Table 1) and also in two previous studies [24,25]. All investigations were performed with informed consent and in accordance with the Declaration of Helsinki, and the study was approved by the Danish Committee System on Biomedical Research Ethics. To be able to determine if mutations in specific domains of *CAPN3 *affect regeneration more than others we included as many homozygous patients with LGMD2A as possible. Thirteen patients were homozygous for mutations in *CAPN3*, and six were functionally homozygous, because one allele was a null allele confirmed in previous studies [26-28]. They found calpain 3 was not detected by western blotting, which is probably due to its degradation by nonsense-mediated mRNA decay. Three patients harbored two null alleles, and were all severely affected. For comparison of the three patients harboring two null alleles (age 48 ± 17 years), we included five clinically matched patients with Becker muscular dystrophy (BMD) (age 31 ± 16 years) and five patients with limb-girdle muscular dystrophy type 2I (LGMD2I) (age 27 ± 12 years) who were compound heterozygous for the p.L276I mutation in the fukutin-related protein gene *FKRP *(Table 1). Diagnoses for the patients with Becker muscular dystrophy were based on deletions in the dystrophin gene were identified in all five BMD patients.

**Table 1:** Genotype, muscle strength, pathological severity, calpain 3 expression and recently regenerating fibers in patients with limb-girdle muscular dystrophy type 2A and 2I, and Becker muscular dystrophy

Patient	Age/ onset	Clinical diagnosis	Mutation	Effect of mutation	GMW	Biopsy origin (force% or MRC)	CK	Calpain 3 WB	nMHC/Vimentin+	Pathological severity	NF	WF	N_fibers_
1M	30/n.d	LGMD2A	p.S48N/p.R748X	Missense/null	0	Q.f. (n.d.)	2400	**≈ **10	0.0%	3	0.0%	0.0%	332

2M	22/11	LGMD2A	p.I178T homoz.	Missense	3	Q.f. (n.d.)	2100	**≈ **0	12.9%	3	0.6%	0.4%	1236

3M	36/35	LGMD2A	p.T184M/c.259-260insT	Missense/null	2	Q.f. (3+)	3000	**≈ **100	0.4%	3	0.0%	0.0%	468

4F	38/28	LGMD2A	p.T192I/p.Y537X	Missense/null	4	Q.f. (n.d.)	900	**≈ **100	0.2%	3	0.0%	0.0%	2804

5M	61/n.d.	LGMD2A	c.643-663del homoz.	In-frame deletion	3	Q.f. (41%)	700	**≈ **20	0.2%	3	0.4%	0.0%	497

6M	41/40	LGMD2A	c.643-663del homoz.	In-frame deletion	3	Q.f. (57%)	600	**≈ **30	2.5%	3	0.3%	0.0%	667

7M	36/32	LGMD2A	p.R289W/p.I661X	Missense/null	3	Q.f. (84%)	5000	**≈ **15	0.2%	3	0.7%	0.0%	601

8M	17/16	LGMD2A	p.V354G homoz.	Missense	4	Q.f. (4)	4600	n.d	17.8%	2	0.4%	0.0%	765

9M	30/30	LGMD2A	p.W373R homoz.	Missense	4	Q.f. (74%)	1400	**≈ **10	11.3%	3	0.2%	0.2%	663

10M	38/22	LGMD2A	p.R437G homoz.	Missense	6	T.a. (25%)	600	**≈ **5	4.6%	3	0.3%	0.4%	1142

11M	43/22	LGMD2A	p.G446S homoz.	Missense	9	Q.f. (36%)	700	**≈ **15	2.9%	1	1.4%	0.7%	1171

12F	46/24	LGMD2A	p.R448H/*c.1992+1G>T*	Missense/null	5	T.b. (n.d.)	500	**≈ **0	3.3%	2	0.1%	0.0%	2195

13F	23/15	LGMD2A	p.N449H homoz.	Missense	4	Q.f. (4+)	2700	**≈ **5	4.8%	2	0.3%	0.0%	1451

14M	44/36	LGMD2A	p.R461C homoz.	Missense	3	Q.f. (49%)	4900	**≈ **55	0.3%	3	0.2%	0.0%	1415

15F	49/47	LGMD2A	p.R490Q homoz.	Missense	5	T.b. (n.d.)	1000	**≈ **100	0.5%	3	0.0%	0.0%	811

16M	16/16	LGMD2A	p.R490Q homoz.	Missense	0	Q.f. (n.d.)	n.d.	**≈ **100	4.7%	3	0.0%	0.0%	2402

17M	38/19	LGMD2A	p.R748Q homoz.	Missense	4	T.b. (4)	n.d.	**≈ **0	4.6%	2	0.1%	0.0%	2202

18F	20/12	LGMD2A	p.I777T/c.550delA	Missense/null	6	B.b. (3)	2300	**≈ **0	17.6%	2	0.2%	0.0%	873

19F	46/22	LGMD2A	p.A798E homoz.	Missense	6	Q.f. (90%)	1600	**≈ **30	2.8%	3	0.6%	0.0%	360

20M	38/9	LGMD2A	*c,1800+2T>C homoz*.	Null	9	T.a. (14%)	200	0	0.3%	1	1.9%	3.1%	483

21M	68/29	LGMD2A	*c.380-13T>A homoz*.	Null	9	Q.f. (0%)	200	0	0.8%	1	2.7%	7.8%	257

22M	38/15	LGMD2A	p.E285X homoz.	Null	9	T.a. (7%)	n.d.	0	0.0%	1	1.7%	6.0%	351

23-27	27 ± 12/6 ± 4	LGMD2I	Various	Missense	9.0 ± 0.0	T.a. 6 ± 6%	700 ± 700	55 ± 20	22.7 ± 12.7%	1.0 ± 0.0	1.7 ± 1.5%	3.4 ± 3.1%	781 ± 372

28-32	34 ± 12/25 ± 11	BMD	Various	Missense	8.6 ± 0.5	T.a. 9 ± 9%	4700 ± 7300	95 ± 5	20.3. ± 13.8%	1.8 ± 0.7	0.8 ± 0.7%	0.7 ± 0.9%	499 ± 402

M/F = male/female; Age = age when biopsy was taken; n.d. = not determined; LGMD2A = limb-girdle muscular dystrophy type 2A; LGMD2I = limb-girdle muscular dystrophy type 2I; BMD = Becker muscular dystrophy; Mutations in introns are marked in italic; GMW = modified Gardner-Medwin-Walton scale; Q.f. = Quadriceps femoris; T.b. = Triceps brachii; B.b. = Biceps brachii; T.a. = Tibialis anterior; CK = creatine kinase; WB = presence of calpain 3 (94 + 60 bands) in western blot in percent of normal; nMHC/Vimentin + = Regenerating fibers expressing neonatal myosin heavy chain and vimentin; Pathological severity: (3 = mild), (2 = moderate) and (1 = severe); NF = Necrotic fibers; WF = Whorled fibers; N_fibers _= number of fibers analyzed in each section

The modified Gardner-Medwin and Walton clinical severity score [29], was used for our patients: grade 0 = hyperCKaemia, all activities normal; grade 1 = normal gait, unable to run freely, myalgia, atrophy; grade 2 = unable to walk on tiptoes, waddling gait; grade 3 = evident muscle weakness, stepping gait, climbing stairs with banister; grade 4 = difficulty in rising from the floor, presence of Gowers' sign; grade 5 = unable to rise from the floor; grade 6 = unable to climb stairs; grade 7 = unable to rise from a chair; grade 8 = unable to walk without assistance; grade 9 = unable to eat, drink or sit unassisted.

### Muscle force

The ankle dorsal flexion and knee extension forces, corresponding to the muscle that was biopsied, were measured by dynamometry [30,31]. Muscle force measurements were compared to the ankle dorsal flexion (196 ± 52 N) and knee extension (269 ± 77 N) forces found in healthy subjects (14 males and 13 females, age 32 ± 12 years). For eleven patients with LGMD2A muscle force measurements corresponding to the muscle that was biopsied at the time of biopsy were not obtainable. However five of them had a clinical evaluation of the biopsied muscle on the MRC scale whereas the remaining six did not have any functional assessment at the time of biopsy (Table 1).

### Muscle *b*iopsy *a*nd *h*istochemistry

**Figure 1:**
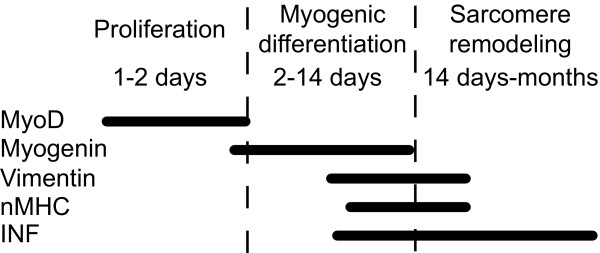
**Sequential presence of regeneration markers MyoD, myogenin, neonatal myosin heavy chain (nMHC), vimentin and internally nucleated fibers (INF) based on previous reports **[18,20-22,32,33].

Percutaneous needle muscle biopsies were obtained as part of the diagnostic procedure and snap-frozen in liquid nitrogen-cooled isopentane, and stored at -80°C. The quadriceps femoris was biopsied in sixteen patients, the tibialis anterior in thirteen and the biceps/triceps brachii in four patients. Ten μm serial cryo-sections were used for all stains. Sections were stained with hematoxylin and eosin for general histopathological evaluation, and assessment of internally nucleated fibers (INF). A myofiber was considered INF if at least one nucleus was non-peripheral. Internal nuclei originate from activated satellite cells, and reside internal for a period of several months after regeneration initiation in skeletal muscle (Figure 1) [19,32,33]. Two investigators (SH and TOK) independently assessed all biopsies blinded for patient data. Complete sections were evaluated for the number of INF and necrotic fibers. In addition we determined number of whorled fibers, representing an irregular regeneration of mature myofibers where the sarcomere alignment in the outer layers is perpendicular to the core [34].

### Pathological severity

Biopsies were graded on a 3-point pathological severity scale based on the dystrophic changes as previously described [2]: 1 = active dystrophic process (marked increase of fiber size variability, active degeneration and regeneration, marked increase of connective tissue); 2 = moderate dystrophic process (marked increase of fiber size variability, increased central nuclei, few degenerating and regenerating fibers, slight increase of connective tissue); 3 = mild myopathic picture (moderate increase of fiber size variability, increased central nuclei).

### Immunohistochemistry

For immunohistochemistry, all sections were, fixed in acetone and methanol (1:1) or 4% paraformaldehyde (for MyoD), and subsequently blocked in buffer (3% fetal calf serum in PBS) prior to staining. Primary antibodies were diluted 1:100. To assess the number of myofibres presently undergoing regeneration, sections were stained with neonatal myosin heavy chain (nMHC) (Vector Laboratories, Burlingame, CA, USA). Muscle fibers showing a faint nMHC reaction (intermediate stain) and nuclear clumps (with positive nMHC staining) were excluded from the calculation, in order to consider only active regenerating fibers. To confirm that fibers expressing nMHC were actually regenerating the adjacent section were stained with the intermediate filament vimentin (clone V9, Novocastra, UK). Both nMHC and vimentin are usually expressed 1-3 weeks after initiation of regeneration (Figure 1) [20,22]. Co-expression of nMHC and vimentin during regeneration has previously been described in patients with muscular dystrophy [35]. Satellite cells were visualized with antibodies against myogenic transcription factors MyoD (Vector Laboratories) targeting activated cells and differentiating cells with myogenin (clone F5D, Developmental Studies Hybridoma Bank, Iowa City, IA, USA). These myogenic markers are expressed within 24 hours and 1-7 days respectively of satellite cell activation (Figure 1) [18,21]. Positive nuclei were confirmed by DAPI nuclear stain (Invitrogen, Carlsbad, CA, USA) and to be in a satellite cell position under the basal lamina by using an antibody against laminin (L9393; Sigma, St Louis, Missouri, USA). Alexa 488 and 594 (Invitrogen, Carlsbad, CA) secondary anti- mouse and anti-goat antibodies were used at a 1:500 dilution in PBS buffer. For detection of apoptosis in sub-sarcolemmal myonuclei, sections were TUNEL-stained using the manufacturer's protocol (Roche Diagnostics, Hvidovre, Denmark), and subsequently stained with cleaved PARP fragment antibody (Biosource, Camarillo, CA, USA), Laminin alpha-2 (clone 22B2, Novocastra, UK) and DAPI nuclear stain. Fibers that were nuclei-positive for all three stains were considered apoptotic [36-38]. The sections were observed under a Nikon 80i microscope with epi-fluorescence.

### Western blot and densitometric analysis

**Figure 2:**
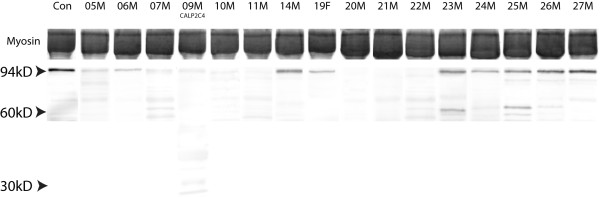
**Western blot used for densitometric analysis of calpain 3 amount using mouse anti-human calpain 3, clone 12A2 (Novocastra) and for patient 9M mouse anti-human calpain 3, clone 2C4 (Novocastra)**. Homogenate from a healthy control muscle (Con) and from patients were loaded and the presence of calpain 3 (94 + 60 bands) of each sample was normalized to the amount of skeletal myosin bands in the post-transfer Coomassie blue-stained gels and quantity of calpain 3 is expressed as percentage of control.

Calpain 3 was analyzed by western blot (Figure 2) using mouse anti-human calpain 3, clone 12A2 (Novocastra) recognizing the full size and 60 kDa bands and clone 2C4 recognizing full size and 30 kDa band as previously described [2]. Lanes of healthy control muscles were loaded on each gel. Dried gels and blots were scanned at 600 dpi on an Epson GT8000 flatbed scanner using white light for gels and blue for blots. Each image was stored as a 16-bit TIFF grayscale and ImageJ v1.41 software was used for the densitometric analysis. The presence of calpain 3 (94 + 60 bands) of each sample was normalized to the amount of skeletal myosin bands in the post-transfer Coomassie blue-stained gels and quantity of calpain 3 is expressed as percentage of control rounded off to nearest 5 percent.

### Genetic analyses

Genetic analysis was performed as described elsewhere [24,25]. Primers and conditions for testing mutations in *FKRP *were performed as described before [39].

### Statistics

Statistical significance among groups was determined using Student's unpaired *T*-test. A significance level of p < 0.05 was considered significant. All numbers provided are mean ± SD. Logarithmic and linear regression analysis was used, and p-value was determined from a Pearson correlation coefficient table of critical values. For pathological severity comparison a Freeman-Halton extension of the Fisher exact probability test for a two-row by three-column contingency table was used.

## Results

### Histology

**Figure 3:**
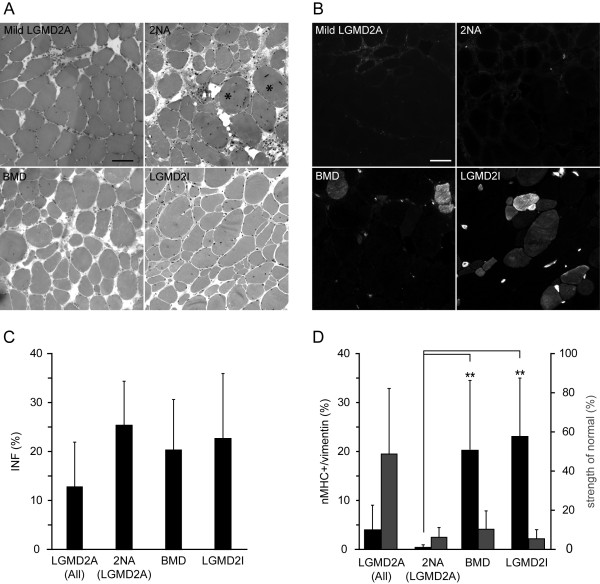
**Hematoxylin & eosin and neonatal myosin heavy chain stains are shown for patients with Limb-Girdle muscular dystrophy type 2A, patients with two *CAPN3 *null alleles (2NA), patients with Becker muscular dystrophy and patients with LGMD2I (A), (B)**. Asterisks denote whorled fibers. Bar is 100 μm. Comparison of internally nucleated fibers (INF) in patients with LGMD2A, those with two *CAPN3 *null alleles (2NA), BMD, and LGMD2I shows no difference among groups **(C)**. In the same patient groups, the relationship between both nMHC and vimentin-positive fibers (black bars) and corresponding muscle strength of biopsied muscle, expressed as percentage of normal, (grey bars) is shown **(D)**.

The muscle morphology from sections stained with H&E showed necrotic fibers, fibrosis and muscle fat infiltration in almost all patients (Table 1; Figure 3A). The three LGMD2A patients with two null-alleles (no. 20-22 in the table) all displayed active dystrophic process; marked increase of fiber size variability, active degeneration and regeneration, marked increase of connective tissue, comparable with that found in muscles from the five compound heterozygous patients with LGMD2I and the five patients with BMD (Table 1; Figure 3A). We found in patients with LGMD2A that the number of INF varied from approximately 2% to 34% of the total number of fibers, mean being 13.1% ± 8.8% (Figure 3C). While muscles with severe dystrophic features in general had a high number of INF's, there were exceptions; one severely affected patient had 10.8% INF's and five mildly affected patients with moderate muscle pathology had 6 to 16% INF's (Table 1). We did not observe any significant difference in INF's among LGMD2A, LGMD2I and BMD patients (Figure 3C).

### Regeneration *m*arkers

**Figure 4:**
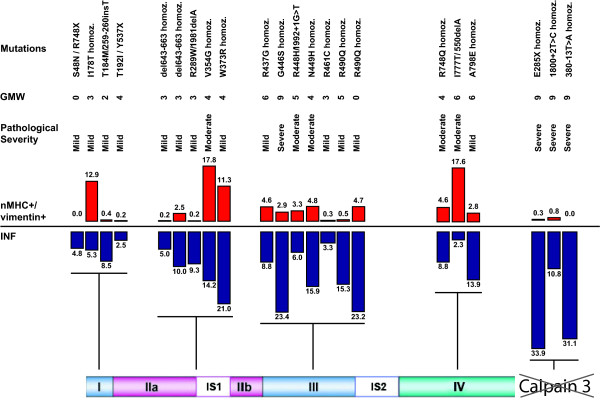
**Distribution of patients with LGMD2A ordered by mutation site and grouped into domains I-IV of CAPN3**. Level of internally nucleated fibers and fibers expressing both neonatal myosin heavy chain and vimentin are displayed with Gardner-Medwin-Walton scale and pathological severity.

**Figure 5:**
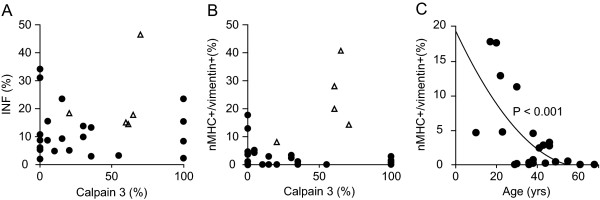
**Regeneration markers, internally nucleated fibers (INF) (A) and fibers expressing the regeneration markers neonatal myosin heavy chain and vimentin (B) showing no correlation with level of calpain 3 in patients with (• LGMD2A) or (Δ LGMD2I)**. Each symbol represents a patient. Correlation between the fibers expressing the regeneration markers neonatal myosin heavy chain and vimentin and age at biopsy (**C**).

In this study fibers expressing nMHC (Figure 3B) was also co-expressing vimentin in 93% of the regenerating fibers, 7% only expressed nMHC and not vimentin, whereas fibers expressing vimentin and not nMHC was less than 0.1% (data not shown). Only fibers co-expressing nMHC and vimentin were defined as recently regenerating fibers. Severely affected *CAPN3 *null-allelic LGMD2A patients (20 M, 21 M and 22 M, Table 1) have a very low number of recently regenerating fibers 0.4 ± 0.4%, whereas severely affected patients with LGMD2I (no. 23-27 in the table) had 22.2 ± 12.7% (*p *< 0.02) and equally severely affected patients with BMD (no. 28-32 in the table) had 20.3 ± 13.8% (*p *< 0.03) (Figure 3D). Only four patients with LGMD2A (2M, 8M, 9M and 18F) had a high percentage (5%<) of recently regenerating fibers (Figure 4). As the *CAPN3 *null-allelic patients hardly had any recently regenerating fibers (Figure 4), we wanted to address whether there was a relationship between calpain 3 levels and regeneration. Two patients (8M and 9M) had mutations in the amino acids recognized by the calpain 3 (Calp3c/12A2) antibody and consequently calpain 3 level from patient 9M were determined using (Calp3c/2C4) antibody, regrettably calpain 3 data from patient 8M was not obtained. Overall regeneration assessed by INF's did not differ with the level of calpain 3 (Figure 5A). No clear correlation between recently regenerating fibers and level of calpain 3 determined by Western blot was observed (Figure 5B). We subsequently stained for two myogenic transcription factors, MyoD and myogenin, to determine if there was a relationship between the levels of these transcription factors and recently regenerating fibers. Neither was detected at any significant level in patients with LGMD2A, while myogenin was detected in satellite cells in 0.7 ± 0.7% of the fibers in patients with LGMD2I, and 0.6 ± 0.3% in patients with BMD consistent with the higher level of recently regenerating fibers in these patients. There was an inverse correlation between number recently regenerating fibers and age at biopsy, which was only significant in patients with LGMD2A (*p *< 0.001) (Figure 5C), whereas number of recently regenerating fibers was not correlated with disease duration (*p *= 0.65).

### Pathological severity

**Figure 6:**
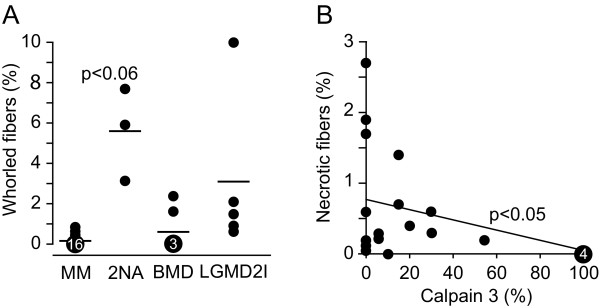
**Whorled fibers in patients with LGMD2A with missense mutations (MM) and those with two null-alleles (2NA)**. Findings are shown as the mean and range (p < 0.06) **(A)**. Necrotic fibers and level of calpain 3 in patients with LGMD2A (p < 0.05) **(B)**.

When comparing the number of whorled fibers in patients with LGMD2A, the three patients with two null-alleles they had a tendency for a higher presence than the other nineteen patients with LGMD2A (Figure 6A) (*p *< 0.06). We found a negative correlation of necrotic fibers with level of calpain 3 (Figure 6B) (*p *< 0.05). Apoptotic nuclei were only found in three patients with LGMD2A, none exceeding 0.2% of the total number of fibers and none found in patients with LGMD2I or BMD (data not shown).

## Discussion

This study suggests that functional calpain 3 is a prerequisite for adequate muscle regeneration, and indicate that part of the pathogenesis in LGMD2A may relate to a deficiency in regenerating damaged muscle fibers. This is based on: Severely affected patients with *CAPN3 *null-alleles, i.e. no functional calpain 3, had almost no recent regeneration and since the clinical and pathological severity of LGMD2A, LGMD2I and BMD is comparable, we can assume that it is the lack of calpain 3 in patients with LGMD2A, and not the severity of the disease, that affects the regenerative process. Our findings are consistent with studies of calpain 3 knockout mice which have decreased levels of embryonic myosin heavy chain and abnormal sarcomere organization [13].

We found that patients with LGMD2A whom did not harbor null-alleles, while having no detectable level of calpain 3, were still able to regenerate their muscles. It is possible that minute quantities of calpain 3, not detectable by Western blotting, still have remaining function shown for a LGMD2A patient homozygous for p.R748Q [40]. This could be sufficient to maintain sarcomere remodeling at a very basic level of muscle regeneration. In addition, function of calpain 3 may not always correspond to the level of calpain 3 protein detected by Western blot, so the mutant protein may be inactive even if it is present [41]. Studies of inactive calpain 3 knock-in mice suggests that calpain 3 may have other functions than acting as a protease and it is possible that calpain 3-dependent muscle regeneration, in particular sarcomere remodeling and incorporation of developmental myosins is based on non-proteolytic functions of calpain 3 rather than the obvious protease [4]. Consistent with this, we have found that severely affected patients with LGMD2I having a secondary loss of calpain 3 protein, while having a high level of nMHC/vimentin positive fibers. While the loss of calpain 3 can be considerable, the residual albeit normal calpain 3 in LGMD2I patients, is apparently sufficient to maintain the regenerative process.

The mechanism by which calpain 3 acts in the regeneration process is unknown but it has been suggested that calpain 3 aids the shift of non-muscle myosin heavy chain in pre-myofibrils to muscle myosin heavy chain during myofibrillogenesis [13]. This implies that calpain 3 deficiency would lead to a prominent decrease in post-fusion incorporation of nMHC, hence the regeneration of the myofiber after damage is attenuated. This is also supported by a study of patients with LGMD2A showing that patients with near normal levels of calpain 3 had slow disease progression, whereas patients with absent or very low quantities of calpain 3 had rapid progression and early onset of weakness [25]. This is consistent with the findings for the *CAPN3 *null-alleles patients, where the absence of calpain 3 as seen in patients with two *CAPN3 *null-alleles has a profound negative impact on the regenerative response, so severely affected patients with LGMD2A with the highest need for regeneration have the most impeded response.

We noted that regeneration among the patients with LGMD2A whom did not harbor null-alleles, had a tendency of age-dependency, consistent with other findings of age negatively affecting muscle regeneration [42,43]. In fact, one common feature among the four patients with the highest level of recent regeneration is that they were among the youngest of all included LGMD2A patients when they were examined and the biopsy was taken. In other words, it appears that in this study the ability to maintain a higher level of recent regeneration is better at a younger age. However, on a grander scale, with young patients having other calpain 3 mutations than what we include in this study, this may change. The three severely affected patients with *CAPN3 *null-alleles in the present study were older than the severely affected patients with LGMD2I and BMD, so it is possible that the regenerative capacity was reduced even further by the increased age.

A key aspect of this study is the fact that muscle regeneration varies tremendously among the patients. One may ask why some mildly affected patients display as much recent regeneration as severely affected patients or why regeneration in terms of INF of mildly affected patients spans from 2.5% to 23.2%. There is no simple answer to these questions, however, we propose that ultimately muscle regeneration is a matter of degeneration and capability. Since the effect of the various mutations on the function of calpain 3 are not well understood, two different patients can experience the same clinical course, even though the mutations may affect different functions of calpain 3. The level and frequency of muscle degeneration may vary to an extent, if a mutation e.g. affects the processing of AHNAK, thus the dysferlin-mediated membrane repair, or a different mutation impedes the sarcomere remodeling. For this reason we have focused on the difference between complete lack of calpain 3 in the calpain 3 null patients and patients having some calpain 3 left affected by various mutations.

The patients with LGMD2A with two *CAPN3 *null-alleles have a high number of INF. Thus, the initial steps of regeneration such as fusion of myoblasts to the damaged myofiber and migration of the nuclei to the center of the fiber, is not affected by loss of functional calpain 3, unlike the subsequent part of myofibrillogenesis [19]. Calpain 3 has been reported to modulate the myogenic factor MyoD in fully differentiated C2C12, thus promoting the formation of reserve cells in these myotubes, which would correspond to satellite cells in vivo [5,44]. In calpain 3 null patients, the repeated regeneration/regeneration cycle could ultimately cause exhaustion of the satellite cell pool. However, we do not have any evidence this is taking place as it would require an in-depth analysis of satellite cell activation in a larger cohort of calpain 3 null patients, due to the fact that activated satellite cells in vivo are only detectable within a few days.

Calpain 3 null patients had a very clear tendency of whorled fibers (p < 0.06), compared to the patients with LGMD2A having missense mutations. Whorled fibers are an obvious indication of sarcomeres in disarray, as part of the fiber contains sarcomeres perpendicular to the fiber direction. While whorled fibers have been described earlier as a feature of LGMD [45], we are not aware of any previous study, which demonstrates this difference in LGMD2A. As whorled fibers are seen in other LGMD and BMD, predominantly severely affected patients, it is clear that whorled fibers is not caused by calpain 3 deficiency, but some yet unknown mechanism likely related to massive degeneration. A study of rabbit skeletal muscle immobilized in the most stretched position found that the whorled fibers represent an irregular regeneration, where the sarcomere alignment in the outer layers is perpendicular to the core [34].

Necrotic fibers, a sign of degeneration, are dependent on the level of calpain 3 in the patients with LGMD2A in our study. While it is difficult to point to one specific reason calpain 3 deficiency causes necrotic fibers, it is possible that in some myofibers the combination of a severed link between calpain 3 and dysferlin-mediated membrane repair and dysfunctional sarcomere remodeling ultimately leads to the demise of the myofiber.

We did not find a significant number of apoptotic myonuclei in patients with LGMD2A, which suggests that myonuclear apoptosis is not a primary feature of calpain 3 deficiency in the patients included in this study. This seems consistent with observations in calpain 3 knockout mouse muscle [13]. However, it has previously been reported that lack of calpain 3 leads to apoptosis [14]. This discrepancy can be attributed to either choice of apoptotic markers or difference in genotype of the patients.

More information from in vitro studies of enzyme activity of mutated calpain 3 is necessary before any correlation between function and phenotype can be made. It is often assumed that all of these mutations reduce/abolish the function of calpain 3, but this is not always the case [6,46]. The null alleles clearly abolish protein function, but missense mutations in *CAPN3 *have unpredictable consequences at protein level [46,47]. This may result in rapid autolysis, thus reducing the function of calpain 3 dramatically.

## Conclusions

Our results indicate that activation of satellite cells is not the limiting step, as presence of INF is independent on the level of calpain 3, but that the capacity for myofibrillogenesis is dependent on functional calpain 3. By quantifying the regenerative response in patients with LGMD2A this study confirms the findings in the calpain 3 knockout mice about pathogenesis of the disease pointing toward a defective sarcomere remodeling. This knowledge may aid in providing a conceptual framework in which to consider novel therapies.

## Abbreviations

LGMD: Limb girdle muscular dystrophy; BMD: Becker muscular dystrophy; nMHC: neonatal myosin heavy chain; *CAPN3*: calpain 3 gene; INF: internally nucleated fibers; FKRP: fukutin-related protein

## Competing interests

The authors declare that they have no competing interests.

## Authors' contributions

SH carried out immunohistochemistry and drafted the manuscript. MD carried out all genetic analyses. MLS and CA obtained the biopsies and performed muscle force evaluation. JV assisted in experimental design and manuscript editing. TOK assisted in assessing pathological severity, experimental design and manuscript editing. All authors have read and approved the final manuscript.

## Pre-publication history

The pre-publication history for this paper can be accessed here:

http://www.biomedcentral.com/1471-2474/13/43/prepub
